# OsCpn60β1 is Essential for Chloroplast Development in Rice (*Oryza sativa* L.)

**DOI:** 10.3390/ijms21114023

**Published:** 2020-06-04

**Authors:** Qingfei Wu, Cheng Zhang, Yue Chen, Kaiyue Zhou, Yihua Zhan, Dean Jiang

**Affiliations:** 1 State Key Laboratory of Plant Physiology and Biochemistry, College of Life Sciences, Zhejiang University, Hangzhou 310058, China; feiqw1234@163.com (Q.W.); 21607005@zju.edu.cn (C.Z.); nbzky@163.com (K.Z.); 21107014@zju.edu.cn (Y.Z.); 2 Institute of Horticulture, Zhejiang Academy of Agricultural Science, Hangzhou 310021, China; earnchen@163.com

**Keywords:** rice, *OsCpn60β1*, chloroplast, proteomics

## Abstract

The chaperonin 60 (Cpn60) protein is of great importance to plants due to its involvement in modulating the folding of numerous chloroplast protein polypeptides. In chloroplasts, Cpn60 is differentiated into two subunit types—Cpn60α and Cpn60β and the rice genome encodes three α and three β plastid chaperonin subunits. However, the functions of Cpn60 family members in rice were poorly understood. In order to investigate the molecular mechanism of *OsCpn60β1*, we attempted to disrupt the *OsCpn60β1* gene by CRISPR/Cas9-mediated targeted mutagenesis in this study. We succeeded in the production of homozygous *OsCpn60β1* knockout rice plants. The *OsCpn60β1* mutant displayed a striking albino leaf phenotype and was seedling lethal. Electron microscopy observation demonstrated that chloroplasts were severely disrupted in the *OsCpn60β1* mutant. In addition, *OsCpn60β1* was located in the chloroplast and *OsCpn60β1* is constitutively expressed in various tissues particularly in the green tissues. The label-free qualitative proteomics showed that photosynthesis-related pathways and ribosomal pathways were significantly inhibited in *OsCpn60β1* mutants. These results indicate that *OsCpn60β1* is essential for chloroplast development in rice.

## 1. Introduction

Most proteins must fold into their native states to attain functional activity. But in the constantly changed cellular environment and under unpredictable environmental stress conditions, newly synthesized proteins are susceptible to misfolding and aggregation, potentially bring dangers to cells. To maintain cellular protein homeostasis, molecular chaperones play an irreplaceable role in promoting protein efficient folding and preventing protein aggregation [1,2,3]. Among them, chaperonins are a critical group of ATP-driven molecular chaperones that widely exist in prokaryotes and eukaryotes [4,5,6]. They form large double-ring complexes consisting of 14–16 subunits and each ring encloses a central cavity that assists in the folding of encapsulating substrate proteins [7,8,9]. Depending on structure and weather co-chaperonin dependence, chaperonins are classified into two distantly related subgroups, group I and group II [10]. Group I chaperonins were found in eubacteria (GroEL), chloroplast (Cpn60) and mitochondria (Hsp60), while group II chaperonins present in archaea (thermosomes) and the eukaryotic cytosol (CCT or TRiC).

The structure and functional mechanism of Group I chaperonins have been extensively investigated in GroEL/GroES complex [11,12,13]. GroEL forms a homo-oligomer protein composed of two stacked heptameric rings, each of which contains seven identical ~57KD subunits. The co-chaperonin, GroES forms a ring of seven ~10KD subunits which functionally interacts with GroEL in the presence of ATP to assist protein folding. And the functional mechanism of GroEL/GroES complex has been elucidated by two models—An asymmetric “bullet” model and a symmetric “football” model [13,14]. As the homolog of GroEL and Hsp60, chloroplast chaperonin Cpn60 was initially found as the Rubisco binding protein that could combine with Rubisco large subunit (rbcL) and participate in the assembly process of Rubisco holoenzyme [15,16,17]. In contrast to bacteria and mitochondria, Cpn60 contains two distinct subunit types, termed Cpn60α and Cpn60β [16,18,19]. Notably, the amino acid sequences of these two subunit types are only about 50% identical, which also share the equal similarity with GroEL [17,18]. Besides, the structure and regulatory mechanism of Cpn60 are much more complicated than GroEL and Hsp60, due to the existence of multiple copies in photosynthetic eukaryotes genomes [20,21]. In vitro, Cpn60αβ hetero-oligomer and Cpn60β homo-oligomer have been reconstructed in *E.coli* cells. In the presence of MgATP, both of these two types of oligomers could display refolding activity when assisted by co-chaperonins in vitro [22]. However, Cpn60β subunits preferentially form Cpn60αβ hetero- oligomers in the presence of Cpn60α. Besides, only hetero-oligomeric Cpn60αβ1β2 other than homo-oligomeric Cpn60β can cooperate functionally with GroES in refolding activity [23]. And in *Chlamydomonas reinhardtii*, both Cpn60 monomers and homo-oligomers possessed ATPase activity; but only protein complexes containing all three subunits, the Cpn60αβ1β2 oligomeric complex, have functional cooperation with GroES in refolding a model substrate [24,25]. These results suggest that in vivo Cpn60αβ hetero-oligomers play a much more important role than homo-oligomers. It has been proposed that Cpn60 hetero-oligomers contain seven α subunits and seven β subunits (Cpn60α7β7) [16,18,26]. However, a recent study found that in *Chlamydomonas reinhardtii*, Cpn60α:Cpn60β1:Cpn60β2 was determined in a 6:2:6 ratio [27], which indicated that the structures of Cpn60 vary significantly among species and need to be further studied.

The molecular mechanism of Cpn60 proteins has been well investigated in *Arabidopsis*. There are two *AtCpn60α* genes (*AtCpn60α1*: *At2g28000*, *AtCpn60α2*: *At5g18820*) and four *AtCpn60β* genes (*AtCpn60β1*:*At1g55490*, *AtCpn60β2*: *At3g13470*, *AtCpn60β3*: *At5g56500*, *AtCpn60β4*: *At1g26230*) in *Arabidopsis* genome. Among them, the expression level of *AtCpn60α1*, *AtCpn60β1* and *AtCpn60β2* is much higher than three others. A T-DNA insertion mutant of *AtCpn60α1*, termed *schlepperless* (*slp*), was the firstly identified mutant of Cpn60 in *Arabidopsis* [28]. The *slp* mutant exhibited an embryonic lethality phenotype due to a defect in plastid development which affected embryo development before the heart stage. Besides, a missense allele (caused by a single nucleotide mutation) of *AtCpn60α1*, *arc2*, showed a dwarf phenotype with reduced chlorophyll contents and chloroplast numbers, which suggested that *AtCpn60α1* was critical to the development of chloroplast and embryo [29]. *AtCpn60α2* mutants showed abnormal embryos arrested at the globular stage, which was possibly caused by the reduction in the KASI (β-ketoacyl-[acyl carrier protein] synthase I) protein level. In contrast to AtCpn60α1, AtCpn60α2 could form functional hetero-oligomers with AtCpn60β2 and AtCpn60β3, which is specifically required for the folding of KASI [30]. A mutant of *AtCpn60β1*, *br04*, showed a growth retardation phenotype with enlarged chloroplasts, while the mutant of *AtCpn60β2* did not exhibit embryo development defects or chloroplast division defects [29]. The *atcpn60β1atcpn60β2* double mutant exhibited albino and seedling lethal phenotypes, suggesting *AtCpn60β1* and *AtCpn60β2* have redundant functions in chloroplast division. AtCpn60β4 can form a hetero-oligomeric complex with AtCpn60α1 and three other AtCpn60β subunits and is essential for the folding of NdhH [31]. Besides, a recent report showed that *AtCpn60β4* also regulates growth, development and flowering in *Arabidopsis* [32].

In rice, there are three OsCpn60α genes (OsCpn60α1: LOC_Os12g17910, OsCpn60α2: LOC_Os03g64210, OsCpn60α3:LOC_Os09g38980) and three OsCpn60β genes (OsCpn60β1: LOC_Os06g02380, OsCpn60β2: LOC_Os02g01280, OsCpn60β3: ChrSy.fgenesh.gene.28). However, few experiments have focused on OsCpn60 and only two of the six family members have been studied in detail. A T-DNA insertion mutant of OsCpn60α1 exhibited pale-green and seedling lethal phenotypes, whose protein level of rbcL was severely reduced, suggesting that OsCpn60α1 is an essential factor for rbcL folding [33]. In addition, the rice gene TCD9 encodes a subunit of chaperonin protein (OsCpn60α2), which is important for chloroplast development during the early leaf stage [34]. In this study, in order to investigate the molecular mechanism of OsCpn60β1, we constructed OsCpn60β1 knockout mutants by CRISPR/Cas9 technology which exhibited albino leaf phenotype. By combining a phenotypic and comparative proteomics analysis, we found that OsCpn60β1 plays an important role in the chloroplast development.

## 2. Results

### 2.1. OsCpn60β1 Deficiency in Rice Results in Albino Leaf Phenotype

**Figure 1:**
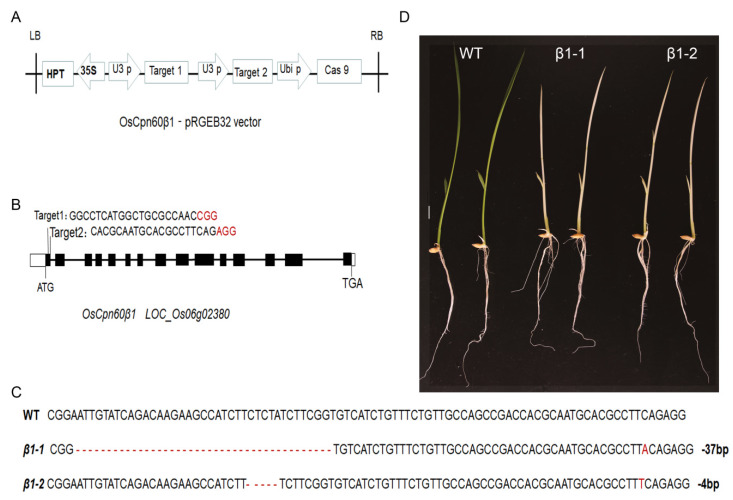
Production of *OsCpn60β1* knockout mutants via the CRISPR/Cas9 system. (**A**) Diagram of CRISPR/Cas9 system for editing *OsCpn60β1*. (**B**) Schematic diagram of targets sites in *OsCpn60β1*. Black boxes show exons, black lines show introns and white boxes show untranslated regions (UTR). (**C**) Mutation sites of *OsCpn60β1* knockout lines. *β1-1* mutant has a 38-bp deletion and a 1-bp insertion, which has a 37-bp deletion in total; *β1-2* mutant has a 5-bp deletion and a 1-bp insertion, which has a 4-bp deletion in total. (**D**) Phenotypes of *OsCpn60β1* mutants, 7-day-old seedlings were photographed. Scale bar, 1cm.

**Figure 2:**
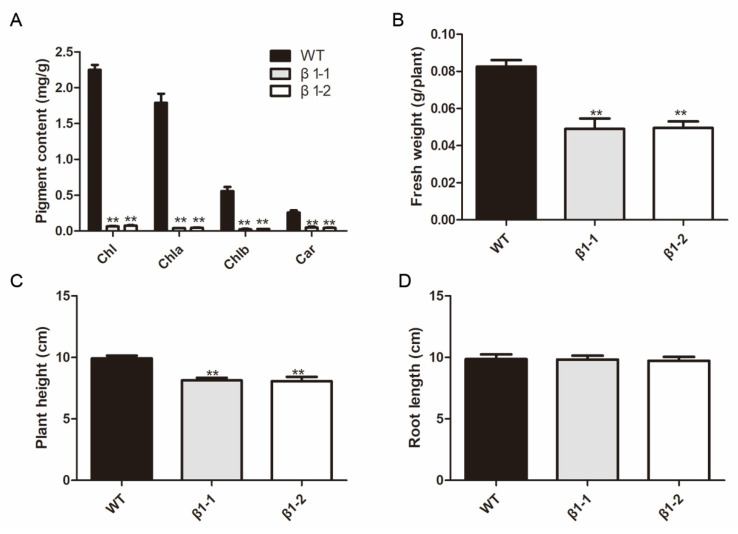
Characteristics of *OsCpn60β1* mutants at 7-day-old seedling stage. (**A**) Pigment content of wild type (WT) and *OsCpn60β1* mutant. Chlorophyll a (Chla), chlorophyll b (Chlb), total chlorophyll (Chl) and carotenoid (Car). (**B**) Fresh weight of WT and *OsCpn60β1* mutants. (**C**) Plant height of wild type and *OsCpn60β1* mutant. (**D**) Root length of WT and *OsCpn60β1* mutants. The data are mean ± SD (n = 3) and ** indicates statistical significance at *p* < 0.01.

In order to clarify the physiological function of *OsCpn60β1*, we attempted to disrupt the *OsCpn60β1* gene by CRISPR/Cas9 genome editing system (Figure 1A). We designed two specific guide RNA sequences in the first exon of *OsCpn60β1* as the editing targets (Figure 1B). And in the T_2_ generation, two transgene-free homozygous knockout lines were recovered after sequencing, which were named *β1-1* and *β1-2* (Figure 1C,D). Both of these tow mutants display albino leaf and growth retardation phenotypes (Figure 1D). Consistent with their phenotypes, the accumulation of chlorophyll was much lower in *OsCpn60β1* mutants than that of wild type (Figure 2A). The seedling fresh weight and plant height of *OsCpn60β1* mutants were much lower compared with wild type (Figure 2B,C), while there were no obvious difference in root length (Figure 2D). Unlike some other albino or chlorotic leaf mutants, who can turn to be green and normal during the later developmental stage [35,36,37,38], the *OsCpn60β1* mutant could not survive past three leaves stage. To examine whether there were other off targets genes in *OsCpn60β1* mutants, we produced the ProCpn60β1::Cpn60β1-GFP (green fluorescent protein) vector construct and transformed into *OsCpn60β1* heterozygous plants. Finally, we obtained 8 independent transgenic lines in the *OsCpn60β1* homozygote background and both of them can rescue the albino leaf phenotype (Figure S1). These results suggest that the phenotypes of the mutant are resulted from *OsCpn60β1* deficiency and *OsCpn60β1* mutants are photosynthesis-deficient mutants in rice.

### 2.2. Chlorophyll Fluorescence Analysis of OsCpn60β1 Mutants

**Figure 3:**
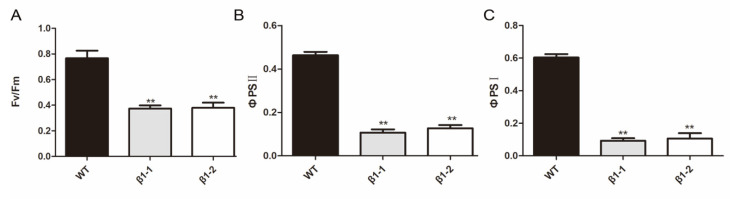
Parameters measurement of WT and *OsCpn60β1* mutants. (**A**) Maximum photochemical efficiency of PSII, Fv/Fm. (**B**) The actual photochemical efficiency of PSII, ФPSII. (**C**) The actual photochemical efficiency of PSI, ФPSI. The data are mean ± SD (n = 3) and ** indicates statistical significance at *p* < 0.01.

Chlorophyll fluorescence is a non-invasive signature of photosynthesis, which has been widely used to monitor changes in the physiological state of the photosynthetic apparatus [39].Therefore, we measured the fluorescence parameters in wild type and the *OsCpn60β1* mutant by a Dual-PAM 100 chlorophyll fluorescence analyzer (Heinz Walz, Effeltrich, Germany) to monitor whether or not the *OsCpn60β1* mutant loss the physiological function. The Fv/Fm ratio reflects the maximum quantum efficiency of photosystem II (PSII) photochemistry and the Fv/Fm was about 0.79 in the wide type plants, whereas the Fv/Fm was just 0.32 in *OsCpn60β1* mutants (Figure 3A). The actual photochemical efficiency (ФPSII and ФPSI) was also reduced dramatically compared with that of wild type, suggesting that the light energy harvest and transfer were severely affected in *OsCpn60β1* mutants (Figure 3B,C).

### 2.3. Chloroplast Development is Disturbed in OsCpn60β1 Mutants

**Figure 4:**
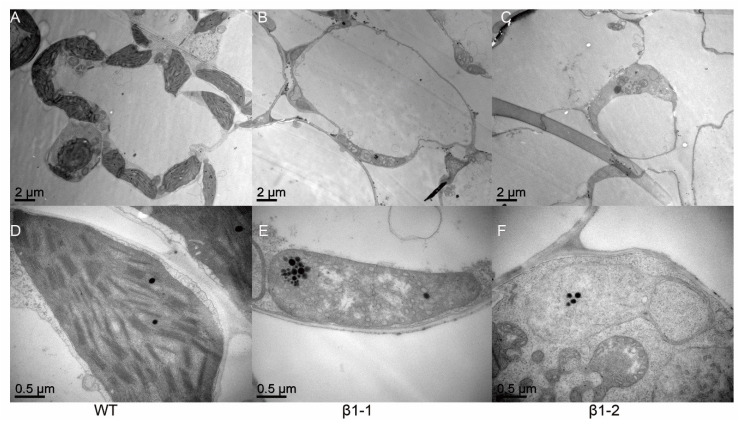
Transmission electron microscopic images of chloroplasts in WT and *OsCpn60β1* mutants. (**A**) The cell of WT; (**B**) the cell of *β1-1* mutant; (**C**) the cell of *β1-2* mutant; (**D**) a normal chloroplast in WT; (**E**) an abnormal chloroplast in *β1-1* mutant; (**F**) an abnormal chloroplast in *β1-2* mutant.

Abnormal chloroplast development should result in lower total chlorophyll contents in plants [40]. To investigate chloroplast development in *OsCpn60β1* mutants further, we observed the ultrastructure of chloroplasts at the three leaves stage of wild type plants and *OsCpn60β1* mutants using transmission electron microscopy (Hitachi H-7650). We observed that the chloroplasts in wild type plants were well developed with dense and well-structured grana stacks (Figure 4A,D). Conversely, the *OsCpn60β1* mutant had abnormal chloroplast architecture without observable grana lamellae stacks, which just displaying oval-shaped vesicles (Figure 4B,C,E,F). These results demonstrated that *OsCpn60β1* plays a key role in early chloroplast development.

### 2.4. Expression Pattern and Subcellular Localization of OsCpn60β1

**Figure 5:**
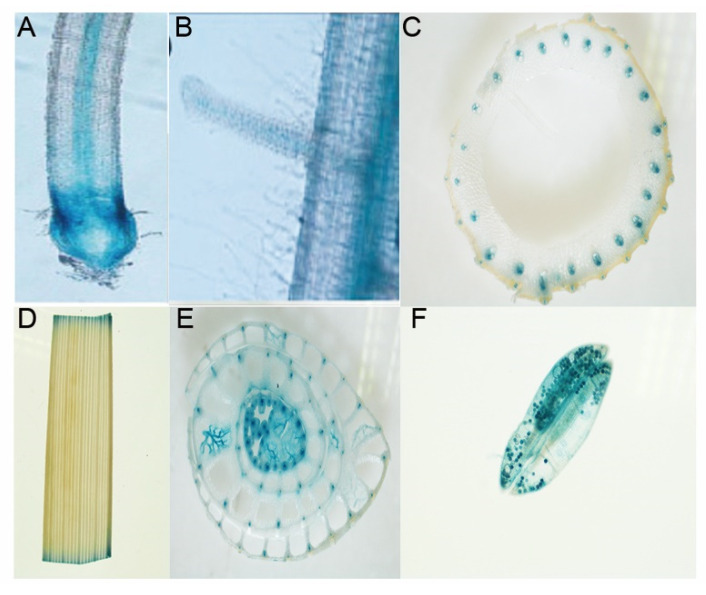
Organic expression pattern of *OsCpn60β1*. Gus staining in axial root (**A**), lateral root (**B**), node (**C**), leaf (**D**), cross section of leaf sheath (**E**), anther (**F**).

To investigate the expression pattern of *OsCpn60β1*, we examined the *OsCpn60β1* expression level in various tissues of wild type rice (Donjin) during different growth stages by qRT-PCR. As shown in Figure S2, *OsCpn60β1* was expressed in root, stem, leaf, root–stem transition region, node, flower, panicle and filling seed. And the expression level in the leaves was higher than that of other tissues, especially in the flag leaf expression was the highest and expression in node and filling seed rarely, indicating that *OsCpn60β1* mainly functions in leaves (Figure S2). To determine the spatiotemporal expression profile of *OsCpn60β1* in detail, we also developed transgenic plants with *OsCpn60β1* promoter driven β-glucuronidase (GUS). Consistent with the results of qRT-PCR, GUS staining was observed in different tissues including axial root, lateral root, leaf, leaf sheath and anther (Figure 5). Taken together, our results indicated that *OsCpn60β1* was constitutively expressed in various tissues particularly in the green tissues.

**Figure 6:**
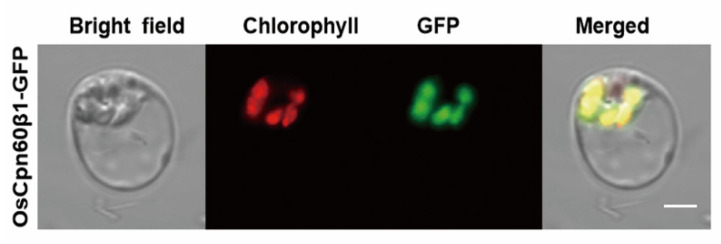
Subcellular localization of the OsCpn60β1-GFP (green fluorescent protein) in rice protoplasts. GFP signals show that the OsCpn60β1-GFP fusion protein produced from the 35S::OsCpn60β1-GFP construct localized to the chloroplast. Green fluorescence shows GFP, red fluorescence shows chloroplast auto- fluorescence and yellow fluorescence shows the merged fluorescence. Scale bar, 5μm.

SignalP (http://www.cbs.dtu.dk/services/SignalP/) [41] analysis of the OsCpn60β1 polypeptide predicted it contains a chloroplast transit peptide. To explore the precise subcellular localization of OsCpn60β1, we produced the 35S::OsCpn60β1-GFP transgene and transiently expressed in rice protoplasts. The green fluorescent signals of OsCpn60β1-GFP were overlaid with chloroplast auto- fluorescence in transformed rice protoplasts (Figure 6). These data demonstrated that OsCpn60β1 was localized in the rice chloroplast.

### 2.5. Analysis of Differentially Accumulated Proteins (DAPs) in OsCpn60β1 Mutants

In order to gain an extensively understanding of the function of *OsCpn60β1*, total proteins in leaves were extracted from the wild type plants and *OsCpn60β1-1* mutants and the proteins expression profiles were detected by using relative liquid chromatography electrospray ionization tandem mass spectrometry(LC-MS/MS-)based label-free protein quantification technique. A total of 3534 proteins were identified using the two biological replicates, with 3168 proteins in wild type plants and 3379 proteins in *OsCpn60β1* mutants. The proteins whose fold change was greater than 2 (up-regulation greater than 2 or down-regulation less than 0.5) and the *p*-value lower than 0.05 were considered as being differentially expressed. Consequently, there were 558 differentially accumulated proteins (DAPs) between wild type and *OsCpn60β1* mutants, among which 277 proteins were up-regulated and 281 were down-regulated in *OsCpn60β1* mutants (Table S1).

**Figure 7:**
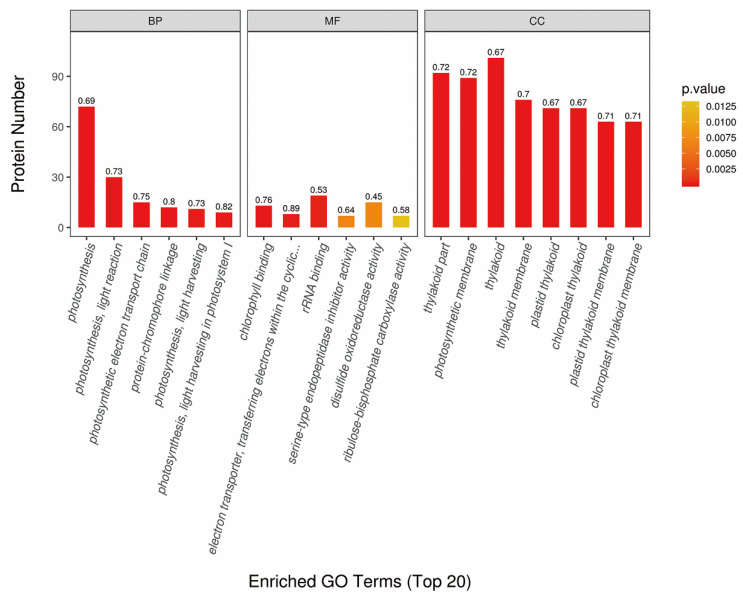
GO functional enrichment analysis of differentially accumulated proteins (DAPs) in *OsCpn60β1* mutants compared with WT. The label at the top of the bar chart shows the enrichment factor (rich Factor ≤ 1), which represents the proportion of the number of DAPs annotated into a GO function category to the number of all identified proteins annotated into the GO function category. The color of the bar chart represents the significance of enriched GO functional classification, which is based on Fisher’s exact test to calculate the P value. BP, biological process; MF, molecular function; CC, cellular components.

The overall identified proteins were then classified into three sets of ontologies including biological process (BP), molecular function (MF), cellular component (CC) based on GO analysis. The identified proteins were mainly involved in such as metabolic process, cellular process, biological regulation, catalytic activity, protein binding, cell and cell parts (Figure S3). Furthermore, using Fisher’s exact test method, the GO functional enrichment analysis of DAPs was performed to understand the changed cellular metabolism in *OsCpn60β1* mutants. For the BP category, photosynthesis related terms such as light reaction, light harvesting and photosynthetic electron transport chain were the most important terms. For the MF category, chlorophyll binding, RNA binding and disulfide oxidoreductase activity were enriched. For the CC category, thylakoid proteins were significantly enriched. And these results suggest that DAPs are mainly primarily involved in light reaction, light harvesting, photosynthetic electron transport chain, rRNA binding, disulfide oxidoreductase activity (Figure 7).

**Figure 8:**
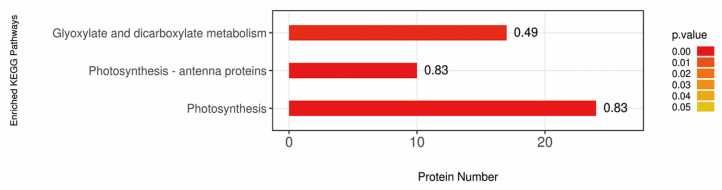
Pathway enrichment analysis of differentially accumulated proteins (DAPs) in *OsCpn60β1* mutants compared with WT. For the bar graph, color represents the significance of enriched kyoto encyclopedia of genes and genomes (KEGG) pathways. Fisher’s exact test is used to calculate the *p*-value. The label at the top of the bar chart shows enrichment factor (rich Factor ≤ 1), which represents the proportion of the number of DAPs involved in a KEGG pathway to the number of proteins involved in this pathway among all identified proteins.

The DAPs were further analyzed for kyoto encyclopedia of genes and genomes (KEGG) pathway enrichment. Using the KEGG database, 558 DAPs were classified into 78 metabolic pathways. According to the numbers of DAPs, the top 20 pathways mainly composed ribosome, photosynthesis, glyoxylate and dicarboxylate metabolism, carbon fixation in photosynthetic organisms and protein processing in endoplasmic reticulum (Figure S4). With Fisher’s exact test, KEGG pathway enrichment analysis of DAPs was performed. And as shown in Figure 8, three pathways were significantly enriched (*p*-value < 0.05), including glyoxylate and dicarboxylate metabolism (17 proteins), photosynthesis (24 proteins) and photosynthesis-antenna proteins (10 proteins).

### 2.6. Photosynthesis Metabolism Proteins Were Greatly Decreased in OsCpn60β1 Mutants

**Figure 9:**
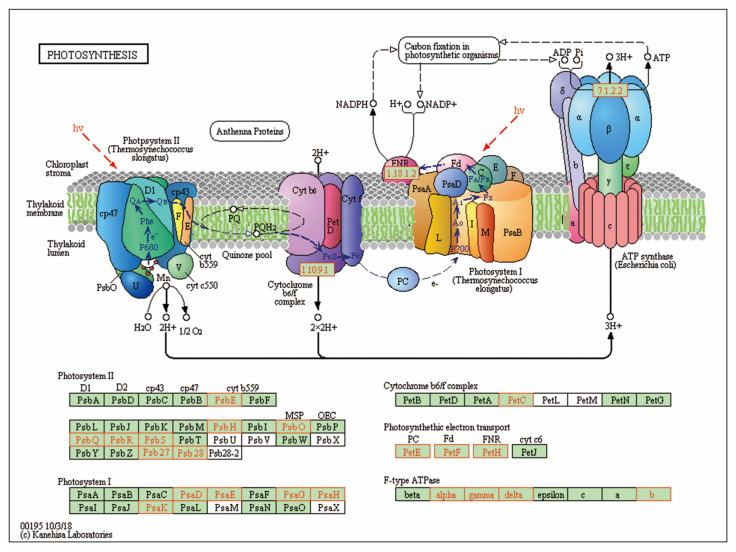
Differentially accumulated proteins (DAPs) involved in photosynthesis pathway. The known pathways were obtained from KEGG database. Red borders indicate differentially accumulated proteins in *OsCpn60β1* mutants. Green background borders indicate proteins which were unidentified identified in WT but not in *OsCpn60β1* mutants. White background borders indicate proteins which were not identified in both WT and *OsCpn60β1* mutants.

As a chloroplast located chaperone, Cpn60 plays an important role in in modulating the folding of numerous chloroplast protein polypeptides, such as rbcL and NdhH [16,31]. The abundance of photosynthesis related proteins contain photosystem I, photosystem II, light harvesting antenna and cytochrome b6/f complex proteins were greatly decreased in *OsCpn60β1* mutants. In Figure 9 is shown the photosynthesis pathway, which had the higher number of associated proteins of all the significantly enriched pathways found in the Fisher’s exact test shown in Figure 8. And 24 proteins were down-regulated significantly (proteins levels were shown in Table S2), including some photosystem II (PSII) complex subunits (such as Psb27, Psb28, PsbE, PsbH, PsbO, PsbQ, PsbR, PsbS), five proteins in photosystem I (PSI) complex (such as PsaD, PsaE, PsaG, PsaH, PsaK), cytochrome b6/f complex (Petc), photosynthetic electron transport proteins (PetE, PetF, PetH) and F-type ATPase (alpha, gamma, delta, b). Light harvesting antenna is one of the most abundant chloroplast proteins in plants, which plays an important role in the absorption, transmission and regulation of excitation energy distribution between photosynthetic reaction centers, as well as in maintaining the stack of the thylakoid membrane [42]. In *OsCpn60β1* mutants, several light harvesting antenna proteins were also down-regulated (Table S2). And these results suggested that light reaction was inhibited seriously in the *OsCpn60β1* mutant. Besides, some proteins participated in the pathway of carbon fixation in photosynthetic organisms were down-regulated seriously (Table S2), especially the abundance of rbcL and Rbcs, which indicated that *OsCpn60β1* is also critical to Calvin cycle regulation.

### 2.7. Ribosomal Pathway Was Inhibited in Oscpn60β1 Mutants

**Figure 10:**
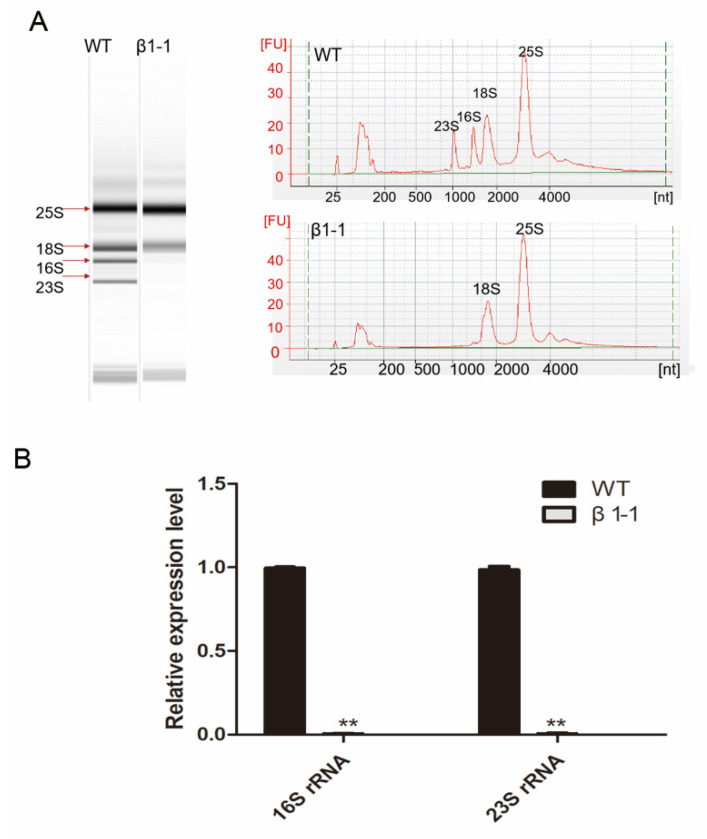
*OsCpn60β1* affects the development of chloroplast ribosome. (**A**) rRNA analysis using an Agilent 2100 bioanalyzer. The rRNAs isolated from 7-day-old WT and *OsCpn60β1* mutants seedlings. (**B**) qRT-PCR analysis of rRNA accumulation in WT and *OsCpn60β1* mutants seedlings. The data are mean ± SD (n = 3) and ** indicates statistical significance at *p* < 0.01.

Ribosomes are the cell’s protein-synthesizing machinery, which comprise a large (60S or 50S) and small (40S or 30S) subunit containing rRNAs and various ribosomal proteins. In eukaryotic cells, ribosomes are found in chloroplast, mitochondria and cytoplasm [43,44]. In the present study, a total of 27 ribosomal proteins were altered significantly in the *OsCpn60β1* mutant compared with wild type, among which except for two 40S ribosomal proteins were up-regulated and the others including most chloroplast ribosomal proteins were down-regulated seriously (Table S3). The chloroplast ribosome includes four rRNA (23S, 16S, 5S and 4.5S) and other ribosomal proteins [45]. We hypothesized that the chloroplast ribosome biosynthesis in the *OsCpn60β1* mutant might be impaired. To prove this hypothesis, we further analyzed the composition and amount of rRNAs using the Agilent 2100 bioanalyzer. In *OsCpn60β1* mutants seedlings, 16S rRNA and 23S rRNA were marginally detected (Figure 10). Overall, the chloroplast ribosome biosynthesis is severely impaired in *OsCpn60β1* mutants.

### 2.8. Validation of Proteomics Data by Parallel Reaction Monitoring (PRM) Method

To confirm the reliability of the proteomics data, 8 DAPs were selected to verify the protein expression levels determined by the PRM method. And the expression trends of these selected proteins were basically consistent with our label-free qualitative proteomics data (Table 1), endorsing that our proteomics data was reliable.

## 3. Discussion

### 3.1. OsCpn60β1 is an Essential Subunit of Cpn60 Complex

In this study, we succeeded in generating *OsCpn60β1* knockout mutants using CRISPR/Cas9 technology, which exhibited albino leaves and eventually died at the seedling stage (Figure 1). However, in *Arabidopsis*, neither a single mutation of *AtCpn60β1* nor *AtCpn60β2* could cause a visible phenotype, only the *atcpn60β1atcpn60β2* double mutant displayed albino and seedling lethal phenotypes, suggesting functions of *AtCpn60β1* and *AtCpn60β2* are redundant in chloroplast division [29]. However, there are three *OsCpn60β* genes in rice, although the phenotypes of *OsCpn60β2* and *OsCpn60β3* are unclear and *OsCpn60β1* plays an irreplaceable role in chloroplast development and plant growth. Besides, the *oscpn60α1* mutant had a pale-green phenotype and development ceased at the seedling stage [33]; *OsCpn60α2* mutation caused albino phenotype at low temperature before three leaves seedling stage [34]. Because the different phenotypes of Cpn60 family members and the seedling lethal trait of *OsCpn60β1* mutants (Figure 1), we could conclude that OsCpn60β1 is an essential subunit of Cpn60 complex, whose functions differ from those of other subunits.

### 3.2. OsCpn60β1 is Critical to Rubisco Folding and Carbon Fixation

In the 1980s, when John Ellis studied light driven-protein synthesis in isolated chloroplasts, he found that before combined with RbcS to form a whole enzyme, rbcL first combined with other proteins to form a complex, which was later widely known as Cpn60 [15,16,17]. Later more studies showed that Cpn60 and rbcL were correlated, especially *Cpn60α* subunit types played an important role in the folding of rbcL. In maize, rbcL was closely bound to two specific Cpn60 subunits, namely *ZmCpn60α1* (*cps2*, encoded by AC215201.3) and *ZmCpn60β1* (GRMZM2G083716) [46]. Compared with the wild type, the *cps2* mutant showed pale-green phenotype and the protein abundance of Rubisco was down-regulated by 95%, while the expression level of some other chloroplast proteins did not change significantly, indicating that *ZmCpn60α1* is specific to rubisco folding [47]. In the same way, when the *ZmCpn60α1* homologous gene in rice (*OsCpn60α1*) was mutated and it also showed a yellowish and Rubisco protein specific down-regulation phenotype [33].

It is interesting to note that the abundance of rbcL is unchanged in four mutants of *Arabidopsis*: *β1*, *β2*, *β1/β2* double and *β4* [29,31]. However, the reconstruction of *Arabidopsis thaliana* Rubisco in *E. coli* had been achieved with co-expression of Cpn60 hetero-oligomers and other co-chaperonins. Meanwhile, AtCpn60β, which forms tetradecamer complexes by itself, can also mediate production of Rubisco with low efficiency [23]. Here, we found that the protein abundance of rbcL and Rbcs was marginally detected in our *OsCpn60β1* mutants according to our proteomic data (Table S2) and we also found some proteins participated in Calvin cycle regulation such as fructose-bisphosphate aldolase, malate dehydrogenase are also down-regulated significantly (Table S2), which demonstrates that *OsCpn60β1* is critical to Rubisco folding and plays an important role in carbon fixation.

### 3.3. OsCpn60β1 is Crucial in Ribosome Biogenesis

Chloroplasts contain 70S ribosomes, consisting of a 30S small subunits and a 50S large subunit, similar to prokaryote-type ribosomes and distinct from the cytosolic ribosomes, which are 80S ribosomes [43]. The 30S subunit contains a 16S rRNA and 24 different proteins. The 50S subunit contains 4 rRNAs (23S, 16S, 5S and 4.5S) and 33 different proteins. In addition, plastid ribosomal proteins (PRPs) play important roles in ribosome biogenesis, plastid protein biosynthesis and chloroplast differentiation [48]. Mutation of PRPs caused diverse phenotypic effects in plants, including lethality, reduced plant height and decreased photosynthetic capacity [49]. The maize photosynthetic mutant *hcf60* exhibited a pale green seedling lethal and high-chlorophyll fluorescence phenotypes, which was caused by the deletion of ribosomal small subunit protein 17 (RPS17) and was the first reported PRP mutant in higher plants [48]. In recent years, numerous PRP large subunits and small subunits have been reported to be essential for embryogenesis in *Arabidopsis* [49,50,51,52]. In addition, when some subunits such as RPS1, RPS17 and RPL24 were disturbed, the plastid protein synthesis and photosynthesis of the mutant were impaired in these mutants but they still could survive normally, which indicates that these subunits may not be essential for basal ribosome activity [49].

In rice, *albino seedling lethality 1* (*asl1*) was the first identified PRP mutant and the mutated *ASL1* gene encodes the chloroplast 30S ribosomal protein S20 (RPS20) [53]. Similarly, the rice mutants *asl2* and *al1* were reported to show albino phenotypes at the seedling stage and could not survive past the three leaves stage, due to lacking of the chloroplast 50S ribosomal protein L21 (PRPL21) and 50S ribosome protein L12 (PRPL12) [54,55]. More recently, PRPL13 and RPS6 were found to be required for normal chloroplast development under low temperature conditions [56]. In addition, PRS9 was essential for early chloroplast development in rice, as the *wgl2* mutant displayed an albino phenotype from germination through the three leaves stage and then gradually turned from albino to green through the later developmental stage [56]. In our study, we found that a total of 27 ribosomal proteins were altered significantly in the *OsCpn60β1* mutant compared with wild type, among which 25 ribosome proteins including many chloroplast ribosomal proteins were down-regulated seriously (Table S3). Ribosomes are the cell’s protein-synthesizing machinery and these down-regulated ribosomal proteins would cause a block in the mutant proteins synthesis especially chloroplast proteins. It is worth to note that two cytosolic ribosomal proteins (40S ribosomal protein S3a and 40S ribosomal protein S29) were up-regulated in mutants (Table S3).And cytosolic ribosomal proteins play important roles in many biological processes such as shoot meristematic function, lateral root initiation and leaf variegation [57,58,59,60], we speculated that the increased abundance of these two proteins were essential for plant growth in *OsCpn60β1* mutants at early leaf stage. And the detailed relationships between OsCpn60β1 and these two cytosolic ribosomal proteins need to be further investigated in the future. Besides, the chloroplast ribosome consists of 4 rRNAs (23S,16S, 5S and 4.5S) and these rRNAs represent essential components of the chloroplast translational apparatus [45]. We detected the composition and content of rRNAs in *OsCpn60β1* mutants and wild type. And we found that 23S and 16S rRNAs were significantly decreased in mutants (Figure 10). These results suggested that *OsCpn60β1* was crucial in chloroplast ribosome biogenesis and defects in chloroplast ribosomes would result in abnormal synthesis of chloroplast proteins, which seemed to be a main cause of albino leaf phenotype.

## 4. Materials and Methods

### 4.1. Plant Materials and Growth Conditions

Cultivar ‘Donjin’ was used in the study. Germinated rice seeds were grown in hydroponic solution recommended by the International Rice Research Institute. Rice seedlings were grown in a greenhouse under a 12-h-light (30 °C)/12-h-dark (22 °C) photoperiod and a photosynthetic photon flux density (PPFD) of 500 μmol photons m^−2^s^−1^ as previously described [61].

### 4.2. Construction of Vectors and the Generation of Transgenic Plants

We employed the CRISPR/Cas9 system to establish *OsCpn60β1* mutants according to the protocol described previously [62]. Briefly, the first coding exon of *OsCpn60β1* was selected for guide RNA design based on the CRISPR-PLANT database (www.genome.arizona.edu/crispr/) [63]. Two Polycistronic tRNA-gRNA (PTG) genes were inserted in the pRGEB32 vector to create the OsCpn60β1-pRGEB32 vector by Golden Gate Assembly. To determine the *OsCpn60β1* expression pattern, a 1800-bp sequence upstream of the ATG codon in the *OsCpn60β1* gene was amplified from genomic DNA of wild type, which was cloned into the pBI101.3 vector to drive expression of the GUS reporter gene. For complementation of the *OsCpn60β1* mutant, the CDS of *OsCpn60β1* driven by its 1800-bp native promoter was inserted into the modified pCambia1300-GFP vector to generate the ProCpn60β1::Cpn60β1-GFP vector. Transgenic rice plants were generated by the *Agrobacterium tumefaciens* strain EHA105–mediated transformation using rice mature seeds derived callus according to a conventional protocol [64]. All of the primers are listed in Table S4.

### 4.3. Chlorophyll Quantification and Chlorophyll Fluorescence Measurements

Leaf chlorophyll content was determined according to the previously described method [65] with slight modifications. Leaves (approximately 0.2 g fresh weight) at 7-day-old seedling stage were cut and immersed in 5 mL of 80% acetone for 12 h in the dark. Residual debris was removed by centrifugation. Absorbance of the supernatants was measured by spectrophotometric scanning (DU800, Beckman, Fullerton, CA, USA) at 663 nm, 645 nm and 470 nm. Three biological replicates were analyzed for each sample.

The chlorophyll fluorescence parameters of wild type plants and *OsCpn60β1* mutants were measured using a Dual-PAM 100 chlorophyll fluorescence analyzer (Heinz Walz, Effeltrich, Germany). Prior to measurements, all plants were first dark adapted for 40 min. The photochemical efficiency (Fv/Fm) and electron transfer quantum efficiency (ФPSII and ФPSI) were recorded and calculated using Dual-PAM 100 software according to the manufacturer’s instructions. The measurement was repeated three times and averaged.

### 4.4. Transmission Electron Microscopy

The transmission electron microscopy analysis was carried out as described previously [66]. Concisely, leaf samples were fixed in 2.5% glutaraldehyde in 0.1 M phosphate buffer (pH 7.2) for 24 h at 4 °C, then further fixed in 1% OsO_4_ overnight at 4 °C. Then, tissues were further dehydrated in in a gradient of ethanol solutions and finally embedded in resin. Ultrathin sections (50 nm) were cut on a Leica EM UC7 ultra-microtome and stained with uranyl acetate. Samples examined with a Hitachi H-7650 (Hitachi, Tokyo, Japan) transmission electron microscope.

### 4.5. Subcellular Localization of OsCpn60β1

To investigate the subcellular localization, we cloned the full-length CDS sequence of *OsCpn60β1* without the termination codon and the fragment was fused into the modified pCambia1300-GFP vector under the CaMV35S promoter. Then the 35S::OsCpn60β1-GFP fusion construct was transformed into rice protoplasts according to a previous study [67]. And images were captured by a florescence microscope (Zeiss LSM710, Zeiss, Jena, Germany). The PCR amplification primers are listed in Table S4.

### 4.6. Histological β-glucuronidase (GUS) Assay

GUS staining was performed according to a standard protocol [68]. Transgenic rice tissues were incubated overnight at 37 °C in GUS staining buffer (0.1 M K_2_HPO_4_ (pH 7.0), 0.1 mM KH_2_PO_4_ (pH 7.0), 5 mM K_3_Fe (CN)_6_, 5 mM K_4_Fe(CN)_6_·3H_2_O, 0.1% Triton X-100, 20% methanol, 1 mg mL^−1^ X-Gluc). After staining, the tissues were soaked in 70% ethanol to remove chlorophyll and surface dyes. Images were captured under a stereomicroscope (Nikon AZ100 microscope, Nikon, Kyoto, Japan).

### 4.7. Protein Extraction and Digestion

The shoots of wild type and *OsCpn60β1* mutants at 7-day-old seedling stage were used for protein extraction. In total, there were 6 samples including 2 independent biological replicates and three technical repetitions. Approximately 500 mg fresh tissue of each sample was ground into powder in liquid nitrogen and then incubated in extraction solution (50 mM DTT, 6 M urea, 1% Protease Inhibitor Cocktail and 0.5 M Tris-HCl, pH 8.0). The suspension was cracked by ultrasonication at 4 °C for 5 min and then incubated for 30 min on ice. Samples were subsequently centrifuged at 13,000 g for 30 min at 4 °C and the supernatant was transferred to a new clean tube and stored at −80 °C. The protein concentration was measured with BCA kit (Thermo Fisher Scientific, Waltham, MA, USA) according to the manufacturer’s instructions.

Protein solution was digested using the FASP procedure as previously described [69]. After being reduced with 10 mM DTT at 37 °C for 1 h, the protein solution was alkylated with 30 mM iodoacetamide at 37 °C for 30 min in darkness. Then, 50 mM NH_4_HCO_3_ was added to dilute the urea agent less than 2M. Finally, the sample was further digested using trypsin (Promega, Madison, Wisconsin, USA) in a 1:50 trypsin-to-protein mass ratio at 37 °C overnight. Reactions were stopped by adding formic acid to a final concentration of 1% and the mixture was desalted by a Zorbax column C18 (Phenomenex, Torrance, CA, US). Then, the peptides were vacuum-dried in a Speed Vac Concentrator (Savant, Thermo Fisher Scientific, Waltham, MA, USA) and redissolved in 0.1% formic acid. The concentration of peptides was measured by spectrophotometric scanning at 280 nm.

### 4.8. Label-free Qualitative Proteomics Analysis

The isolation and analysis of tryptic peptides were performed using a quadrupole time-of-flight mass spectrometer (Agilent model 6500, Wilmington, DE, USA) based on the operation manual of MALDI-TOF MS. The resulting MS/MS data were searched against the UniProt plant protein database (http://www.uniprot.org) [70] and the phytozome database (https://phytozome.jgi.doe.gov/pz/portal.html) [71] using the MaxQuant 1.5.3.30 (Computational Systems Biochemistry, Max-Planck Institute for Biochemistry, Martinsried, Germany) software (http://www.coxdocs.org) [72]. The following conditions were used—the enzyme digestion mode was set to Trypsin/P, allowing for up to 2 missing cleavages; the mass tolerance for precursor ions was set at 20 ppm in the first search and at 5 ppm in the main search and the mass tolerance for fragment ions was set at 0.02 Da; carbamidomethyl-modified cysteine residues were specified as a fixed modification and oxidation of methionine was specified as a variable modification. Protein quantitation was calculated using intensity-based absolute quantification (iBAQ) method in MaxQuant (Computational Systems Biochemistry, Max-Planck Institute for Biochemistry, Martinsried, Germany) software, with the *p*-values ≤ 0.05 and the global false discovery rate (FDR) ≤ 0.05. The iBAQ data was weighted and normalized by the median ratio in Mascot. Proteins with a fold change ≥ 2 coupled with *p*-values < 0.05 were determined as differentially accumulated proteins (DAPs).

The bioinformatics analysis was performed according to the method reported in our earlier study [73]. The Gene Ontology (GO) annotation proteome was derived from the UniProt-GOA database (http://www.ebi.ac.uk/GOA/) [74]. Firstly, identified protein IDs were converted to UniProt ID and then mapped to GO IDs by the protein ID. If some identified proteins are not annotated by UniProt-GOA database, the InterProScan software (http://www.ebi.ac.uk/interpro) [75] was used to annotate protein’s GO functional by protein sequence alignment method. GO items can be classified into three categories including cellular component (CC), biological process (BP) and molecular function (MF). For each category, a two-tailed Fisher’s exact test was carried out to test the significance of the enrichment of each differentially accumulated protein (DAPs) against all identified proteins. A GO term with a corrected *p*-value < 0.05 was considered to be significant. The Kyoto Encyclopedia of Genes and Genomes (KEGG) database (https://www.kegg.jp/) [76] was used to annotate the biological pathway. In brief, we got the protein’s KEGG database descriptions by KEGG online service tools KAAS firstly and then mapped the DAPs to the KEGG pathway database by using the KEGG mapper tool.

### 4.9. Parallel Reaction Monitoring (PRM) Verification

In order to confirm the reliability of label-free quantitative proteomics analysis, some DAPs were analyzed by using parallel reaction monitoring (PRM) method. The remaining samples of precious proteomics were used for direct tryptic digestion. The PRM analyses were performed on a mass spectrometer and the peptide fragments monitored for each protein were selected depended on the ion signal intensities in the spectral library. The MS acquisition mode was a combination of two scan events—a full scan and a time-scheduled scan. The full MS scan was carried out with a resolution of 70,000 (at 200 *m*/*z*), an AGC target value of 3.0×10^−6^ and a maximum ion injection time of 250 ms. The time-scheduled scan was taken at a resolution of 35,000 resolution (at 200 *m*/*z*), an AGC target value of 3.0 × 10^−6^ and a maximum ion injection time of 200 ms. And a 2 Th (Thomson) window was used for target precursor ions isolation. Precursor ions were fragmented by HCD (higher-energy collisional dissociation) with normalized collision energy of 27. After each precursor ion (light and heavy masses) was selected by the fragmented quadrupole, all fragment ions were quantified in the orbitrap. Skyline software (MacCoss Lab, Department of Genome Sciences, University of Washington, Seattle, USA) was used to analyze the raw proteomics data and estimate peptide signal intensity. The PRM analysis included three biological replicates.

### 4.10. RNA Extraction and qRT-PCR

Total RNA was extracted using a TaKaRa MiniBEST Plant RNA Extraction Kit according to the manufacturer’s instructions (TaKaRa, Kyoto, Japan). First-strand cDNA was synthesized from 1 μg total RNA using a PrimeScript™RT Master Mix (Perfect Real Time) Kit (TaKaRa, Japan). The qRT-PCR was performed with a TB Green Premix Ex Taq II (Tli RNaseH Plus) Kit (TaKaRa, Japan) using a LightCycler480 instrument (Roche, Basel, Switzerland). The qRT-PCR procedure was as follows—5 min at 95 °C followed by 40 cycles of 95 °C for 10 s and 58 °C for 1 min. Relative expression levels were normalized to that of an internal control, ACTIN (*LOC_Os03g50885*). Fold change of expression was calculated using the 2^−ΔΔCT^ method. The experiment was biologically repeated three times and technically repeated three times for each group. All qRT-PCR primers are listed in Table S4.

### 4.11. rRNA Analysis

Total RNA was isolated from the second leaves of 7-day-old wild type and *OsCpn60β1* mutants seedlings. The concentrations and purities of RNA samples were measured using a NanoDrop Spectrometer (Thermo Fisher Scientific, Waltham, MA, USA). Then RNAs were diluted to 10 ng μL^−1^ and analyzed with an Agilent 2100 bioanalyzer (Agilent Technologies, USA). An RNA 6000 Nano Total RNA Analysis Kit (Agilgent) was used for analysis according to the manufacturer’s instructions.

## 5. Conclusions

In this study, we succeeded in generating homozygous *OsCpn60β1* knockout rice mutants by CRISPR/Cas9 genome editing system. The *OsCpn60β1* mutant exhibited a striking albino leaf phenotype and could not survive past three leaves stage. Compared with wild type plant, the *OsCpn60β1* mutant seedlings had decreased chlorophyll contents, lower fresh weight and plant height. And the *OsCpn60β1* mutant had abnormal chloroplast architecture without observable grana lamellae stacks. In addition, *OsCpn60β1* was located in the chloroplast and constitutively expressed in various tissues. The label-free qualitative proteomics showed that photosynthesis-related pathways and ribosomal pathways were significantly inhibited in *OsCpn60β1* mutants. These results demonstrate that *OsCpn60β1* is critical to chloroplast development in rice.

## Figures and Tables

**Table 1:** Confirmation of Daps in Proteomic Analysis Using parallel reaction monitoring (PRM) Analysis.

Protein ID	Description	Fold Changes in Proteomics (Mutant/WT)	Fold Changes in PRM (Mutant/WT)
Q7XDY9	Rubisco accumulation factor 1	0.452	0.473
Q9ZST1	30S ribosomal protein S17	0.234	0.212
Q6Z7V2	24.1 kDa heat shock protein, mitochondrial	15.022	14.833
Q6H4L2	Elongation factor 2	2.907	2.722
Q7F8S5	Peroxiredoxin-2E-2, chloroplastic	0.329	0.313
Q2QQ99	Protein SPIRAL1-like 3	2.256	2.051
B7E5F1	Carbonic anhydrase	0.061	0.064
Q7F2L7	PsbE	0.016	0.012

## Supplementary Materials

The following are available online at https://www.mdpi.com/1422-0067/21/11/4023/s1: Figure S1. Characteristics of complementation lines at 7-day-old seedling stage. (A) Phenotypes of complementation lines. (B) qRT-PCR analysis of *OsCpn60β1* expression in complementation lines. (C) Pigment content of WT and complementation lines.COM1 represents complementation line 1 of the *OsCpn60β1* mutant, COM2 represents complementation line 2 of the *OsCpn60β1* mutant. Figure S2. Organic expression analysis of *OsCpn60β1* by qRT-PCR. Relative expression level of *OsCpn60β1* was calculated in each tissue of Donjin (DJ) at different growth stages. DJ was cultivated in normal culture solution for 3 weeks and transferred to the field. Figure S3. The GO annotation results of identified proteins in WT and *OsCpn60β1* mutants. Figure S4. The first 20 KEGG pathways with the most DAPs in *OsCpn60β1* mutants compared with WT. Table S1 All DAPs in mutants were listed. Table S2 DAPs in photosynthesis metabolism pathway. Table S3 DAPs in ribosomal pathway. Table S4 Primers sequence in this study were listed.

## Abbreviations

WT	Wild type
GFP	Green fluorescent protein
UTR	Untranslated regions
LC-MS/MS	Liquid chromatography electrospray ionization tandem mass spectrometry
DAPs	Differentially accumulated proteins
GO	Gene ontology
CC	Cellular component
BP	Biological process
MF	Molecular function
KEGG	Kyoto encyclopedia of genes and genomes
TEM	Transmission electron microscopy
PRM	Parallel reaction monitoring
DTT	Dithiothreitol
PSII	Photosystem II
PSI	Photosystem I
RbcL	Ribulose bisphosphate carboxylase large chain
Rbcs	Ribulose bisphosphate carboxylase small chain

## References

[1] Ellis R.J. (1994). Molecular chaperones: Opening and closing the Anfinsen cage. Curr. Biol..

[2] Fenton W.A., Horwich A.L. (2003). Chaperonin-mediated protein folding: Fate of substrate polypeptide. Q. Rev. Biophys..

[3] Hartl F.U., Bracher A., Hayer-Hartl M. (2011). Molecular chaperones in protein folding and proteostasis. Nature.

[4] Bukau B., Horwich A.L. (1998). The Hsp70 and Hsp60 chaperone machines. Cell.

[5] Ditzel L., Löwe J., Stock D., Stetter K.O., Huber H., Huber R., Steinbacher S. (1998). Crystal structure of the thermosome, the archaeal chaperonin and homolog of CCT. Cell.

[6] Sigler P.B., Xu Z., Rye H.S., Burston S.G., Fenton W.A., Horwich A.L. (1998). Structure and function in GroEL-mediated protein folding. Annu. Rev. Biochem..

[7] Leitner A., Joachimiak L.A., Bracher A., Mönkemeyer L., Walzthoeni T., Chen B., Pechmann S., Holmes S., Cong Y., Ma B. (2012). The molecular architecture of the eukaryotic chaperonin TRiC/CCT. Structure.

[8] Xu Z., Horwich A.L., Sigler P.B. (1997). The crystal structure of the asymmetric GroEL-GroES-(ADP)7 chaperonin complex. Nature.

[9] Yébenes H., Mesa P., Muñoz I.G., Montoya G., Valpuesta J.M. (2011). Chaperonins: Two rings for folding. Trends Biochem. Sci..

[10] Horwich A.L., Fenton W.A., Chapman E., Farr G.W. (2007). Two families of chaperonin: Physiology and mechanism. Annu. Rev. Cell Dev. Biol..

[11] Fei X., Ye X., LaRonde N.A., Lorimer G.H. (2014). Formation and structures of GroEL:GroES2 chaperonin footballs, the protein-folding functional form. Proc. Natl. Acad. Sci. USA.

[12] Koike-Takeshita A., Arakawa T., Taguchi H., Shimamura T. (2014). Crystal structure of a symmetric football-shaped GroEL:GroES2-ATP14 complex determined at 3.8A reveals rearrangement between two GroEL rings. J. Mol. Biol..

[13] Nisemblat S., Yaniv O., Parnas A., Frolow F., Azem A. (2015). Crystal structure of the human mitochondrial chaperonin symmetrical football complex. Proc. Natl. Acad. Sci. USA.

[14] Yang D., Ye X., Lorimer G.H. (2013). Symmetric GroEL:GroES2 complexes are the protein-folding functional form of the chaperonin nanomachine. Proc. Natl. Acad. Sci. USA.

[15] Barraclough R., Ellis R.J. (1980). Protein synthesis in chloroplasts. IX. Assembly of newly-synthesized large subunits into ribulose bisphosphate carboxylase in isolated intact pea chloroplasts. Biochim. Biophys. Acta.

[16] Hemmingsen S.M., Ellis R.J. (1986). Purification and properties of ribulosebisphosphate carboxylase large subunit binding protein. Plant Physiol..

[17] Hemmingsen S.M., Woolford C., van der Vies S.M., Tilly K., Dennis D.T., Georgopoulos C.P., Hendrix R.W., Ellis R.J. (1988). Homologous plant and bacterial proteins chaperone oligomeric protein assembly. Nature.

[18] Martel R., Cloney L.P., Pelcher L.E., Hemmingsen S.M. (1990). Unique composition of plastid chaperonin-60: Alpha and beta polypeptide-encoding genes are highly divergent. Gene.

[19] Nishio K., Hirohashi T., Nakai M. (1999). Chloroplast chaperonins: Evidence for heterogeneous assembly of alpha and beta Cpn60 polypeptides into a chaperonin oligomer. Biochem. Biophys. Res. Commun..

[20] Hill J.E., Hemmingsen S.M. (2001). *Arabidopsis thaliana* type I and II chaperonins. Cell Stress Chaperones.

[21] Schroda M. (2004). The Chlamydomonas genome reveals its secrets: Chaperone genes and the potential roles of their gene products in the chloroplast. Photosynth Res..

[22] Vitlin A., Weiss C., Demishtein-Zohary K., Rasouly A., Levin D., Pisanty-Farchi O., Breiman A., Azem A. (2011). Chloroplast β chaperonins from A. thaliana function with endogenous cpn10 homologs in vitro. Plant Mol. Biol..

[23] Aigner H., Wilson R.H. (2017). Plant Rubisco assembly in E. coli with five chloroplast chaperones including BSD2. Science.

[24] Bai C., Guo P., Zhao Q., Lv Z., Zhang S., Gao F., Gao L., Wang Y., Tian Z., Wang J. (2015). Protomer roles in chloroplast chaperonin assembly and function. Mol. Plant.

[25] Zhao Q., Liu C. (2017). Chloroplast chaperonin: An intricate protein folding machine for photosynthesis. Front. Mol. Biosci..

[26] Bonk M., Tadros M., Vandekerckhove J., Al-Babili S., Beyer P. (1996). Purification and characterization of chaperonin 60 and heat-shock protein 70 from chromoplasts of *Narcissus pseudonarcissus*. Plant Physiol..

[27] Zhao Q., Zhang X., Sommer F., Ta N., Wang N., Schroda M., Cong Y., Liu C. (2019). Hetero-oligomeric CPN60 resembles highly symmetric group-I chaperonin structure revealed by Cryo-EM. Plant J..

[28] Apuya N.R., Yadegari R., Fischer R.L., Harada J.J., Zimmerman J.L., Goldberg R.B. (2001). The Arabidopsis embryo mutant schlepperless has a defect in the chaperonin-60alpha gene. Plant Physiol..

[29] Suzuki K., Nakanishi H., Bower J., Yoder D.W., Osteryoung K.W., Miyagishima S.Y. (2009). Plastid chaperonin proteins Cpn60 alpha and Cpn60 beta are required for plastid division in *Arabidopsis thaliana*. BMC Plant Biol..

[30] Ke X., Zou W., Ren Y., Wang Z., Li J., Wu X., Zhao J. (2017). Functional divergence of chloroplast Cpn60alpha subunits during Arabidopsis embryo development. PLoS Genet..

[31] Peng L., Fukao Y., Myouga F., Motohashi R., Shinozaki K., Shikanai T. (2011). A chaperonin subunit with unique structures is essential for folding of a specific substrate. PLoS Biol..

[32] Tiwari L.D., Grover A. (2019). Cpn60*β*4 protein regulates growth and developmental cycling and has bearing on flowering time in *Arabidopsis thaliana* plants. Plant Sci..

[33] Kim S.R., Yang J.I., An G. (2013). OsCpn60α1, encoding the plastid chaperonin 60α subunit, is essential for folding of rbcL. Mol. Cells.

[34] Jiang Q., Mei J., Gong X.D., Xu J.L., Zhang J.H., Teng S., Lin D.Z., Dong Y.J. (2014). Importance of the rice TCD9 encoding α subunit of chaperonin protein 60 (Cpn60α) for the chloroplast development during the early leaf stage. Plant Sci..

[35] Cao Z.L., Yu Q.B., Sun Y., Lu Y., Cui Y.L., Yang Z.N. (2011). A point mutation in the pentatricopeptide repeat motif of the AtECB2 protein causes delayed chloroplast development. J. Integr. Plant Biol..

[36] Gong X., Su Q., Lin D., Jiang Q., Xu J., Zhang J., Teng S., Dong Y. (2014). The rice OsV4 encoding a novel pentatricopeptide repeat protein is required for chloroplast development during the early leaf stage under cold stress. J. Integr. Plant Biol..

[37] Hu Z., Xu F., Guan L., Qian P., Liu Y., Zhang H., Huang Y., Hou S. (2014). The tetratricopeptide repeat-containing protein slow green1 is required for chloroplast development in *Arabidopsis*. J. Exp. Bot..

[38] Yu Q.B., Jiang Y., Chong K., Yang Z.N. (2009). AtECB2, a pentatricopeptide repeat protein, is required for chloroplast transcript accD RNA editing and early chloroplast biogenesis in *Arabidopsis thaliana*. Plant J..

[39] Baker N.R. (2008). Chlorophyll fluorescence: A probe of photosynthesis in vivo. Annu. Rev. Plant Biol..

[40] Sakamoto W., Uno Y., Zhang Q., Miura E., Kato Y., Sodmergen (2009). Arrested differentiation of proplastids into chloroplasts in variegated leaves characterized by plastid ultrastructure and nucleoid morphology. Plant Cell Physiol..

[41] Emanuelsson O., Brunak S., von Heijne G., Nielsen H. (2007). Locating proteins in the cell using TargetP, SignalP and related tools. Nat. Protoc..

[42] Dittami S.M., Michel G., Collen J., Boyen C., Tonon T. (2010). Chlorophyll-binding proteins revisited—A multigenic family of light-harvesting and stress proteins from a brown algal perspective. BMC Evol. Biol..

[43] Subramanian A.R. (1985). The ribosome: Its evolutionary diversity and the functional role of one of its components. Essays Biochem..

[44] Zhang J., Yuan H., Yang Y., Fish T., Lyi S.M., Thannhauser T.W., Zhang L., Li L. (2016). Plastid ribosomal protein S5 is involved in photosynthesis, plant development, and cold stress tolerance in Arabidopsis. J. Exp. Bot..

[45] Tiller N., Bock R. (2014). The translational apparatus of plastids and its role in plant development. Mol. Plant.

[46] Feiz L., Williams-Carrier R., Wostrikoff K., Belcher S., Barkan A., Stern D.B. (2012). Ribulose-1,5-bis-phosphate carboxylase/oxygenase accumulation factor1 is required for holoenzyme assembly in maize. Plant Cell.

[47] Barkan A. (1993). Nuclear mutants of maize with defects in chloroplast polysome assembly have altered chloroplast RNA metabolism. Plant Cell.

[48] Schultes N.P., Sawers R.J., Brutnell T.P., Krueger R.W. (2000). Maize high chlorophyll fluorescent 60 mutation is caused by an Ac disruption of the gene encoding the chloroplast ribosomal small subunit protein 17. Plant J..

[49] Romani I., Tadini L., Rossi F., Masiero S., Pribil M., Jahns P., Kater M., Leister D., Pesaresi P. (2012). Versatile roles of *Arabidopsis* plastid ribosomal proteins in plant growth and development. Plant J..

[50] Bryant N., Lloyd J., Sweeney C., Myouga F., Meinke D. (2011). Identification of nuclear genes encoding chloroplast-localized proteins required for embryo development in *Arabidopsis*. Plant Physiol..

[51] Muralla R., Lloyd J., Meinke D. (2011). Molecular foundations of reproductive lethality in Arabidopsis thaliana. PLoS ONE.

[52] Yin T., Pan G., Liu H., Wu J., Li Y., Zhao Z., Fu T., Zhou Y. (2012). The chloroplast ribosomal protein L21 gene is essential for plastid development and embryogenesis in *Arabidopsis*. Planta.

[53] Gong X., Jiang Q., Xu J., Zhang J., Teng S., Lin D., Dong Y. (2013). Disruption of the rice plastid ribosomal protein s20 leads to chloroplast developmental defects and seedling lethality. G3 Genes Genomes Genet..

[54] Lin D., Jiang Q., Zheng K., Chen S., Zhou H., Gong X., Xu J., Teng S., Dong Y. (2015). Mutation of the rice ASL2 gene encoding plastid ribosomal protein L21 causes chloroplast developmental defects and seedling death. Plant Biol..

[55] Zhao D.S., Zhang C.Q., Li Q.F., Yang Q.Q., Gu M.H., Liu Q.Q. (2016). A residue substitution in the plastid ribosomal protein L12/AL1 produces defective plastid ribosome and causes early seedling lethality in rice. Plant Mol. Biol..

[56] Qiu Z., Chen D., He L., Zhang S., Yang Z., Zhang Y., Wang Z., Ren D., Qian Q., Guo L. (2018). The rice white green leaf 2 gene causes defects in chloroplast development and affects the plastid ribosomal protein S9. Rice.

[57] Byrne M.E. (2009). A role for the ribosome in development. Trends Plant Sci..

[58] Stirnberg P., Liu J.P., Ward S., Kendall S.L., Leyser O. (2012). Mutation of the cytosolic ribosomal protein-encoding RPS10B gene affects shoot meristematic function in Arabidopsis. BMC Plant Biol..

[59] Wang R., Zhao J., Jia M., Xu N., Liang S., Shao J., Qi Y., Liu X., An L., Yu F. (2018). Balance between Cytosolic and Chloroplast Translation Affects Leaf Variegation. Plant Physiol..

[60] Williams M.E., Sussex I.M. (1995). Developmental regulation of ribosomal protein L16 genes in *Arabidopsis thaliana*. Plant J..

[61] Wang D., Li X.F., Zhou Z.J., Feng X.P., Yang W.J., Jiang D.A. (2010). Two Rubisco activase isoforms may play different roles in photosynthetic heat acclimation in the rice plant. Physiol. Plant..

[62] Xie K., Minkenberg B., Yang Y. (2015). Boosting CRISPR/Cas9 multiplex editing capability with the endogenous tRNA-processing system. Proc Natl. Acad. Sci. USA.

[63] Xie K., Zhang J., Yang Y. (2014). Genome-wide prediction of highly specific guide RNA spacers for CRISPR-Cas9-mediated genome editing in model plants and major crops. Mol. Plant.

[64] Hiei Y., Ohta S., Komari T., Kumashiro T. (1994). Efficient transformation of rice (*Oryza sativa* L.) mediated by *Agrobacterium* and sequence analysis of the boundaries of the T-DNA. Plant J..

[65] Wellburn A.R. (1994). The Spectral Determination of Chlorophylls a and b, as well as Total Carotenoids, Using Various Solvents with Spectrophotometers of Different Resolution. J. Plant Physiol..

[66] Guo H., Hong C., Chen X., Xu Y., Liu Y., Jiang D., Zheng B. (2016). Different Growth and Physiological Responses to Cadmium of the Three Miscanthus Species. PLoS ONE.

[67] Yu C., Wang L., Chen C., He C., Hu J., Zhu Y., Huang W. (2014). Protoplast: A more efficient system to study nucleo-cytoplasmic interactions. Biochem. Biophys. Res. Commun..

[68] Jefferson R.A., Kavanagh T.A., Bevan M.W. (1987). GUS fusions: Beta-glucuronidase as a sensitive and versatile gene fusion marker in higher plants. EMBO J..

[69] Wiśniewski J.R., Zougman A., Mann M. (2009). Combination of FASP and StageTip-based fractionation allows in-depth analysis of the hippocampal membrane proteome. J. Proteome Res..

[70] Apweiler R., Bairoch A., Wu C.H., Barker W.C., Boeckmann B., Ferro S., Gasteiger E., Huang H., Lopez R., Magrane M. (2004). UniProt: The Universal Protein knowledgebase. Nucleic Acids Res..

[71] Goodstein D.M., Shu S., Howson R., Neupane R., Hayes R.D., Fazo J., Mitros T., Dirks W., Hellsten U., Putnam N. (2012). Phytozome: A comparative platform for green plant genomics. Nucleic Acids Res..

[72] Cox J., Matic I., Hilger M., Nagaraj N., Selbach M., Olsen J.V., Mann M. (2009). A practical guide to the MaxQuant computational platform for SILAC-based quantitative proteomics. Nat. Protoc..

[73] Zhan Y., Wu Q., Chen Y., Tang M., Sun C., Sun J., Yu C. (2019). Comparative proteomic analysis of okra (*Abelmoschus esculentus* L.) seedlings under salt stress. BMC Genom..

[74] Camon E., Magrane M., Barrell D., Lee V., Dimmer E., Maslen J., Binns D., Harte N., Lopez R., Apweiler R. (2004). The Gene Ontology Annotation (GOA) Database: Sharing knowledge in Uniprot with Gene Ontology. Nucleic Acids Res..

[75] Zdobnov E.M., Apweiler R. (2001). InterProScan--an integration platform for the signature-recognition methods in InterPro. Bioinformatics.

[76] Ogata H., Goto S., Sato K., Fujibuchi W., Bono H., Kanehisa M. (1999). KEGG: Kyoto Encyclopedia of Genes and Genomes. Nucleic Acids Res..

