# Supplementary figures and images for: OsCpn60β1 is Essential for Chloroplast Development in Rice (Oryza sativa L.)
Source: Int J Mol Sci. 2020 Jun 4;21(11):4023. doi: 10.3390/ijms21114023 (PMC7313468; doi:10.3390/ijms21114023)

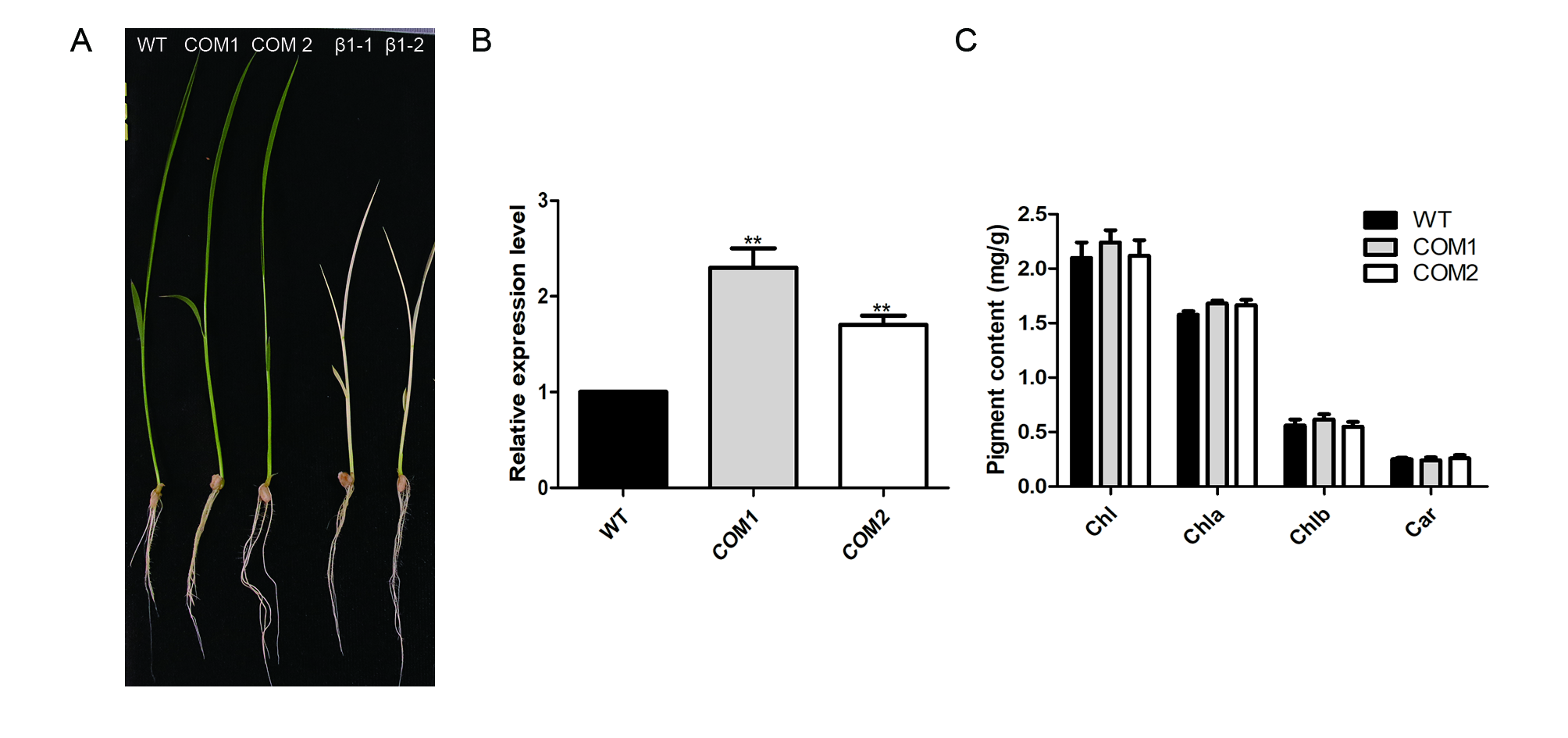

Supplement: Supplementary file 1 [file ijms-21-04023-s001.zip › Supplementary materia/Figure S1.Characteristics of complementation lines at 7-day-old seedling stage..tif]

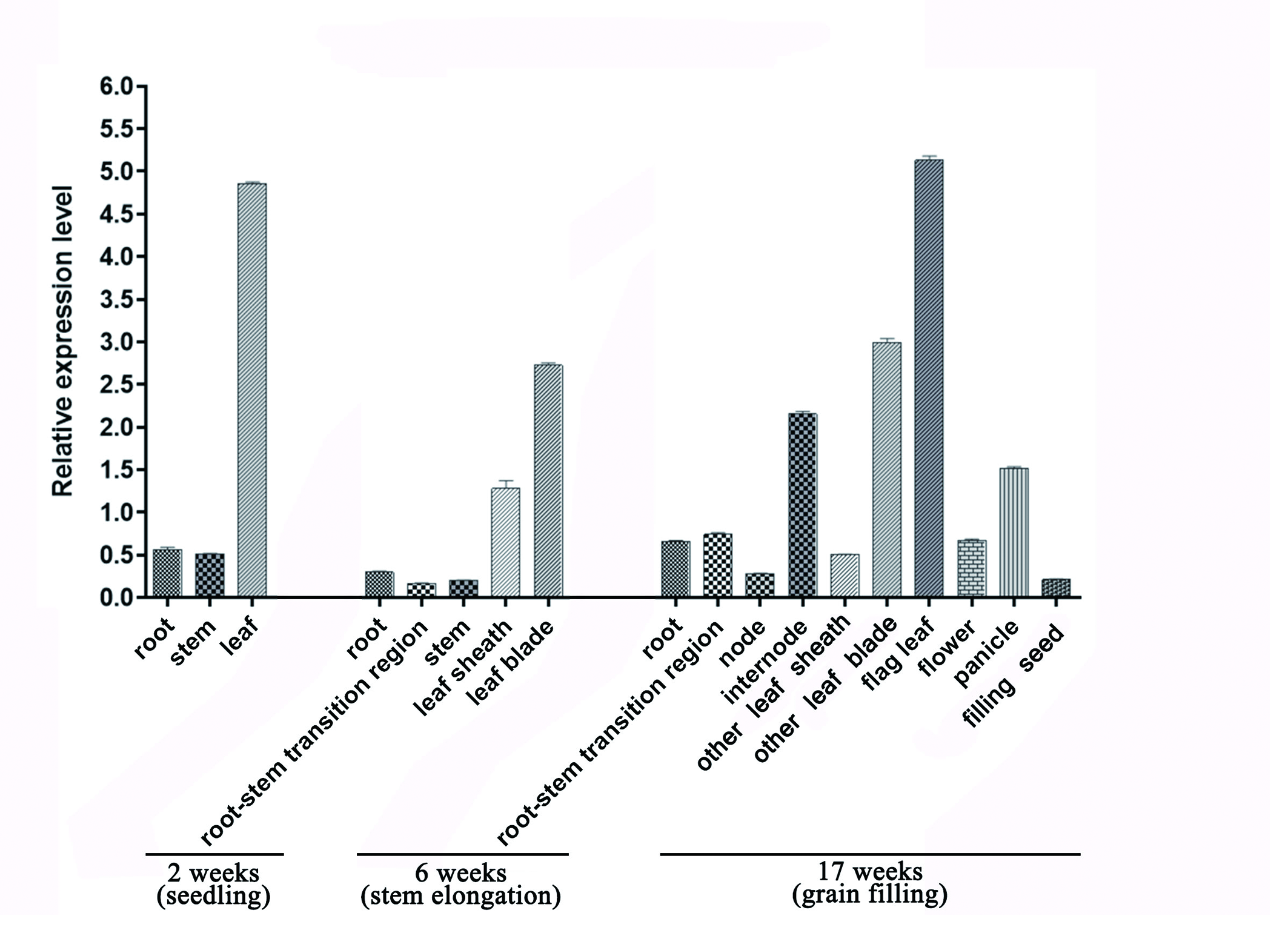

Supplement: Supplementary file 1 [file ijms-21-04023-s001.zip › Supplementary materia/Figure S2. Organic expression analysis of OsCpn6060a┬1 by RT-PCR analysis..tif]

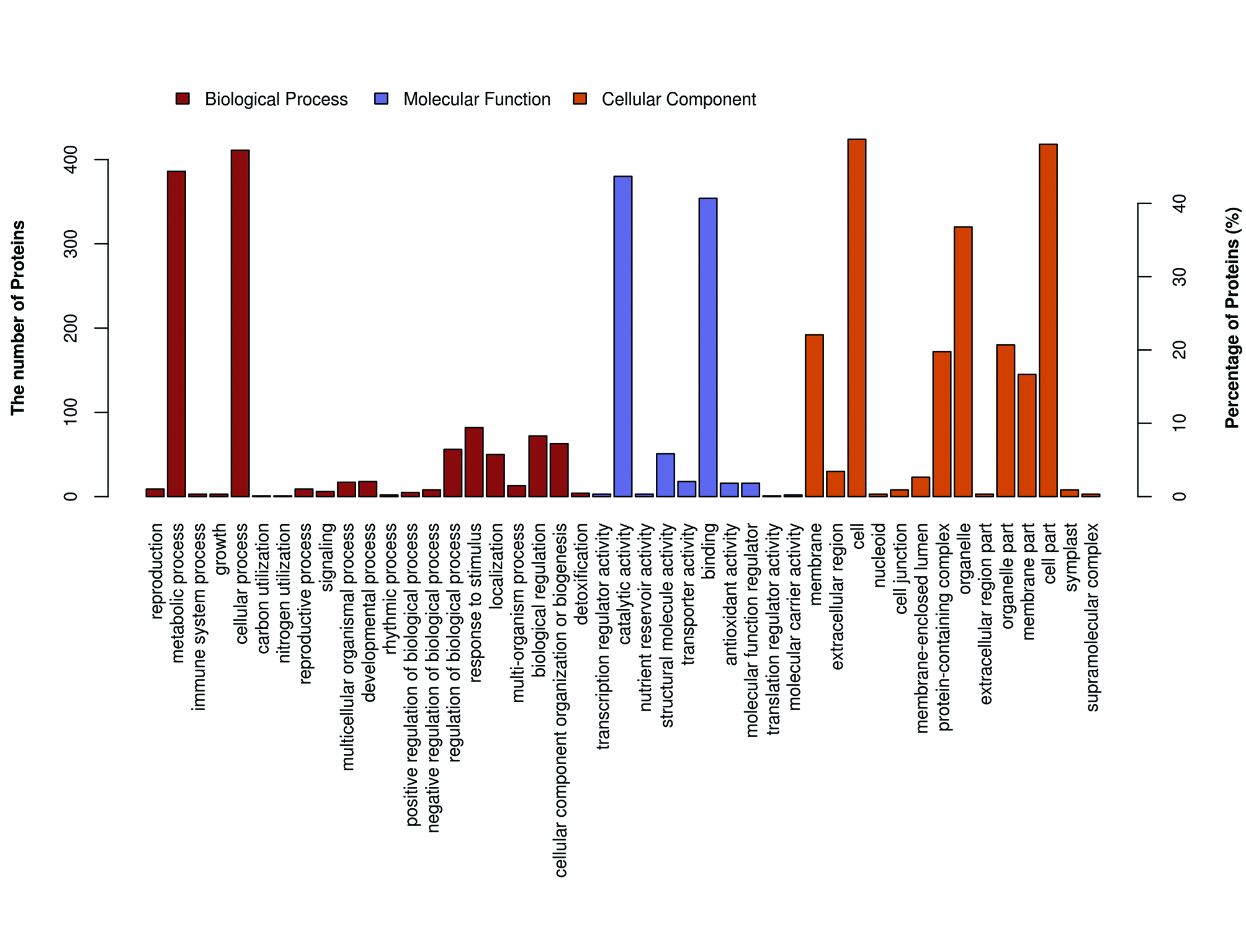

Supplement: Supplementary file 1 [file ijms-21-04023-s001.zip › Supplementary materia/Figure S3. The GO annotation results of identified proteins in WT and oscpn60a┬1 mutants..tif]

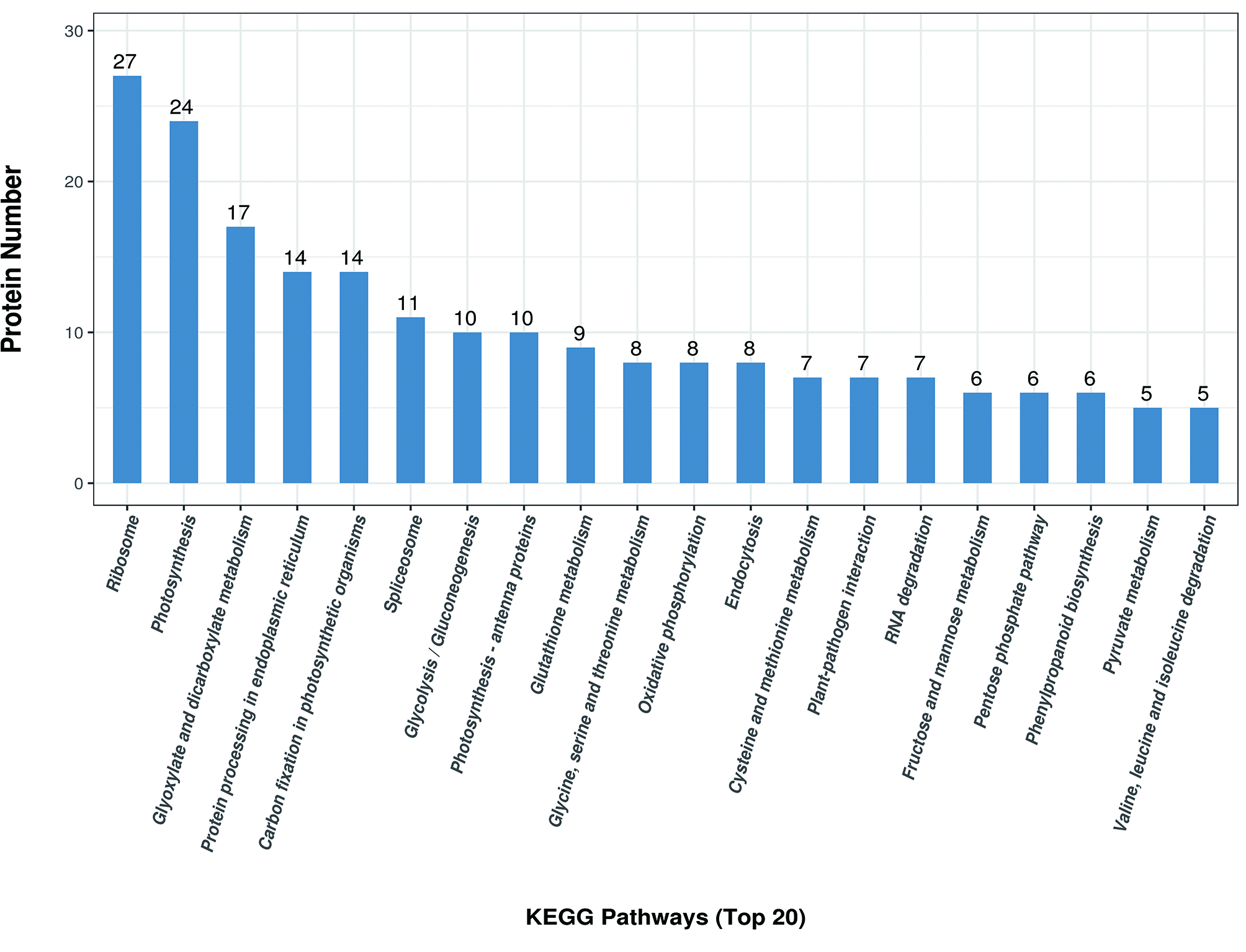

Supplement: Supplementary file 1 [file ijms-21-04023-s001.zip › Supplementary materia/Figure S4. The first 20 KEGG pathways with the most DAPs in oscpn60a┬1 mutants compared with WT..tif]
